# Downgraded dreams: Labor market outcomes and mental health in undocumented migration

**DOI:** 10.1016/j.ssmph.2024.101652

**Published:** 2024-03-06

**Authors:** Carlo Devillanova, Cristina Franco, Anna Spada

**Affiliations:** a Department of Social and Political Sciences and Dondena Centre for Research on Social Dynamics and Public Policy, Bocconi University, Italy; b European Commission, Directorate-General for Neighbourhood Policy and Enlargement Negotiations, Belgium; c On Behalf of Naga, Organizzazione di Volontariato per l’Assistenza Socio-Sanitaria e per i Diritti di Cittadini Stranieri, Rom e Sinti, Italy

## Abstract

Undocumented immigrant workers are particularly exposed to mental health risk factors, including occupational downgrading – i.e. the loss in occupational status upon arrival. This study breaks new ground by examining the relationship between occupational downgrading and mental health among this hard-to-reach population, offering the first-ever investigation of its kind. Leveraging a unique dataset collected by a primary care outpatient clinic in Milan, Italy, which combines medical evaluations with detailed occupational information, we construct a direct measure of occupational downgrading, which adds to the literature. We employ logistic regression models to estimate odds ratios (ORs) for mental and behavioral disorders. The study also offers fresh evidence on the socioeconomic and health status of a sizable sample of undocumented migrants.

The study sample consists of 1738 individuals that had their first medical examination in 2017–18. Prevalence of mental health conditions is 5.58%. Data also highlight poor labor market integration: one third of individuals in the sample is employed, mostly in elementary occupations; 66.63% of immigrant workers experienced occupational downgrading. Regression results show that undocumented immigrants who undergo occupational downgrading are at considerably higher risk of mental disorders. ORs range from 1.729 (95% CI 1.071–2.793), when the model only includes individual characteristics determined prior to migration, to 2.659 (CI 1.342–5.271), when it accounts for all the available controls.

From a policy perspective, our study underscores the need to consider the broader impact of policies, including restrictive entry and integration policies, on migrant health. Additionally, ensuring access to primary care for all immigrants is crucial for early detection and treatment of mental health conditions.

## Highlights

• New evidence on health status and demographics of undocumented migrants.
• Occupational downgrading increases mental health risks among undocumented immigrants.
• Low employment and low-status occupations are prevalent among undocumented migrants.
• Importance of considering the impact of restrictive entry and integration policies on migrant health.

## 1 Introduction

There are approximately 281 million international migrants worldwide, accounting for roughly 3.6% of the global population (McAuliffe & Triandafyllidou, 2021). Among them, an estimated 15%–20% are irregular migrants (Spencer & Triandafyllidou, 2022) – defined as non-nationals who enter or reside in a country without proper documentation (IOM, 2019). Our focus is on those who live in a country without the appropriate documentation (undocumented migrants, henceforth). This highly heterogeneous group of migrants comprises illegal entrants who failed to access regularization, overstayers of visas or work permits and rejected asylum seekers (Spencer & Triandafyllidou, 2022). Despite being at the core of the policy dialogue globally, garnering significant media and public attention, information about this hard-to-reach population remains severely limited in terms of presence, sociodemographic characteristics, and their health status and access to healthcare (Allegri et al., 2022; Fakhoury et al., 2021, 2022; Jackson et al., 2018). Notably, there is a dearth of quantitative evidence on the mental health of undocumented migrants and its determinants, especially in the context of Europe (Fakhoury et al., 2021, Fakhoury et al., 2021).

This knowledge gap is concerning, given that mental disorders are a significant global health burden, with mental health recently incorporated into the Sustainable Development Goals (WHO, 2023b). The World Health Organization (WHO) reports that approximately 280 million individuals worldwide suffer from depression (WHO, 2023a) and predicts that it will become the leading global health issue by 2030 (Malhi & Mann, 2018). Additionally, undocumented immigrants are particularly exposed to mental health risk factors due to the particularly precarious conditions they live and work in (Abbas et al., 2018; Aragona et al., 2013; Castañeda, 2009; Garcini et al., 2016; Moyce & Schenker, 2018). One aspect that has been overlooked so far is that, being undocumented, the vast majority of them undergo significant occupational downgrading, defined in the context of international migration as the “loss in occupational status between one's last job in the home country and first job in the host country” (Crollard et al., 2012, p. 497). To the best of our knowledge, the mental health consequences of occupational downgrading within the context of irregular immigration have not yet been explored. To be sure, the issue has been researched looking at international migrants as a whole. However, existing studies, reviewed in the next section, generally do not observe immigrant workers' occupational downgrading and proxy it with some measure of skill mismatch at destination, hence removing the intrinsically dynamic nature of the concept.

This study, thus, makes two main contributions to the literature. First, it assesses the association between occupational downgrading and mental health among undocumented migrants by leveraging a uniquely large sample of migrants who sought care at an outpatient clinic situated in Milan, Italy. Second, the dataset includes a medical assessment of the primary diagnosis, and it also provides detailed information on occupation in their home country and Italy, thus enabling us to construct a direct measure of worker's occupational downgrading upon arrival. We estimate odd ratios (ORs) through logistic regression models of the probability of mental and behavioral disorders, controlling for a relatively large set of other individual characteristics. As a byproduct, the study offers a relevant insight into the socioeconomic and health status of a sizable sample of undocumented migrants that can be of interest, given the lack of accurate demographic and health data on this population.

Our analysis uncovers a significant link between occupational downgrading and heightened mental health risks among undocumented immigrants. Furthermore, the results document poor integration outcomes within the sample, in terms of employment and quality of occupations.

### 1.1 Occupational downgrading and mental health in international migration

To better grasp the link between occupational downgrading and mental health in international migration, it useful to briefly refer to the substantial literature on the socioeconomic determinants of mental conditions (Allen et al., 2014), among which occupational status features prominently (Alegría et al., 2018). In the native population, unemployment and unfavorable work conditions have been consistently linked to mental and behavioral disorders (Balogh et al., 2023; Barnay, 2016; Rönnblad et al., 2019). Research has also highlighted a negative association between mental health and psychological wellbeing on one hand, and over-education and skills underutilization on the other (Bracke et al., 2013; Chen & Li, 2009; Dean & Wilson, 2009; Milner et al., 2017). Recent evidence further reveals that changes in working conditions over time have a causal impact on mental health outcomes (Belloni et al., 2022). The mental effects of low-quality employment trajectories appear more pronounced among second-generation immigrants (Pollack et al., 2022). Islam and Jaffee (2024) present meta-analyses of the associations between different measures of social mobility, including occupational trajectories, and mental health in the general population, limiting the analyses to studies that did not primarily sample migrant groups.

For international migrants, the association between changes in working conditions over time and mental health turns out to be particularly relevant, given that they are at a high risk of occupational downgrading (Crollard et al., 2012) and loss of social status (Mucci et al., 2019) upon arrival. International migrants often find themselves at an increased risk of occupational mismatch and tend to be clustered in poorly regulated and highly hazardous sectors (Dustmann et al., 2016; Quinlan, 2015). They are more likely to engage in precarious (Zhang et al., 2022) and informal work (ILO, 2016b) and to face on-the-job illnesses and injuries (Moyce & Schenker, 2018; Pega et al., 2021; Sterud et al., 2018). In essence, migrant workers are disproportionately engaged in what are referred to as “3-D jobs” — dirty, dangerous, and demanding (Moyce & Schenker, 2018). Prolonged mismatch status can act as an additional psychosocial stressor for immigrants (Crollard et al., 2012), making them more susceptible to chronic stress and mental health disorders (Bas-Sarmiento et al., 2017; Moyce & Schenker, 2018). Migrants who experienced downward mobility or underemployment were shown to be more likely to screen positive for common mental disorders (Das-Munshi et al., 2012).

As occupational downgrading is an inherently dynamic concept, it is expected to affect immigrant workers' mental health beyond and above the unfavorable working conditions they face. Furthermore, from an analytical perspective, the association between occupational downgrading and mental health is more likely to reflect a causal effect of the former on the latter. Indeed, the main causes of immigrant workers’ occupational downgrading outlined in the literature, including issues like lack of language proficiency, recognition of foreign qualifications, information about local labor market conditions, job opportunities, and discrimination (Aleksynska & Tritah, 2013; Chiswick & Miller, 2009; Dancygier & Laitin, 2014; European Centre for the Development of Vocational Training, 2018), are largely exogenous to individual health conditions.

However, recent research barely observes changes in occupational status upon arrival. Rather, it focuses on the mental health effects of “working below skill level” (Espinoza-Castro et al., 2018), implicitly assuming that over-qualification among international migrants signals some degree of occupational downgrading. These studies consistently find a significant association between mental issues and over-education (Bracke et al., 2013; Dean & Wilson, 2009; Dunlavy et al., 2016), over-qualification (Chen & Li, 2009; Espinoza-Castro et al., 2018; Reid, 2012), poor working conditions (Espinoza-Castro et al., 2021). Two recent review papers further support to the positive association between migrants’ mental health conditions and precarious employment (Koseoglu Ornek et al., 2022) and under-employment (Mucci et al., 2019).

### 1.2 Undocumented immigrants’ mental health

In the context of international migration, undocumented immigrants are particularly exposed to mental health risk factors at different stages of their migration process: while still in their home country, during transit, and upon reaching their destination (Abbas et al., 2018; Aragona et al., 2013; Garcini et al., 2016). The nature of traumatic events preceding arrival in destination countries varies based on their origin, migration motives – especially critical for forced migrants – and the duration and challenges encountered during the journey, including conditions in transit countries, experience of torture and abuses, and the legality of border crossings (Aragona et al., 2023; Fakhoury et al., 2021). Upon arrival at their destination, those individuals grapple with what is referred to as the “illegal syndrome” (Castañeda, 2009), stemming from the constant and chronic stressors they encounter (Garcini et al., 2021). In general, they are not eligible to work legally and frequently end up being exploited in the black-market economy, often in dangerous work environments, where they can seldom validate their formal qualifications and often undergo significant occupational downgrading (Jossen, 2018). They are more likely to remain unemployed, with detrimental effects on their fragile socioeconomic situation, including unstable housing conditions, and are a target of discrimination (Ortega-Jiménez et al., 2023). They constantly live under the threat of deportation due to immigration status-related issues, perpetuating a state of ongoing uncertainty. Additionally, undocumented immigrants may face barriers to accessing basic civil and social rights, including healthcare services (Aragona et al., 2023; Vollebregt et al., 2022; Winters et al., 2018). The fear of deportation may lead some undocumented migrants to abstain from seeking medical attention (Castañeda, 2009; Fakhoury et al., 2021). As such, undocumented migrants are more susceptible to experiencing negative health outcomes in general, and mental health issues in particular (Moyce & Schenker, 2018).

In general, quantitative research on undocumented immigrant health and access to health care presents considerable methodological limitations, such as inadequate sample size, poor outcome measures, and selection bias in recruitment (Allegri et al., 2022; Garcini et al., 2021). Evidence suggests a high prevalence of mental health conditions among undocumented immigrants (Andersson et al., 2018; De Vito et al., 2015; Garcini et al., 2017, Garcini et al., 2021; Kuehne et al., 2015; Martin & Sashidharan, 2023; Patler & Laster Pirtle, 2018; Venkataramani et al., 2017; Vollebregt et al., 2022), although with exceptions (Jackson et al., 2018).

Factors influencing mental health include adverse life events (Aragona et al., 2013; Vollebregt et al., 2022), age and discrimination (Garcini et al., 2017), unstable housing (Andersson et al., 2018). Younger age, social isolation, exposure to abuse, financial instability and multi-morbidity are associated with increased risk of having altered mental health (Fakhoury et al., 2021). Additionally, undocumented migrants comprise rejected asylum seekers that remain in the country, and these individuals are found to be at greater risk of mental illness (Delilovic et al., 2023), also as a result of the mental health consequences of detention policies implemented by many countries (Von Werthern et al., 2018). Importantly, recent evidence shows that immigrants’ legal status represents a mental health risk factor per se (Refle et al., 2023).

### 1.3 Setting

The present study is based in Milan, the capital of Lombardy and second largest Italian city, with 1.4 million inhabitants (3.2 million in the Metropolitan Area). According to the most recent data from the Lombardy Region, as of July 2021 in the Metropolitan Area of Milan, live around 532,800 immigrants, with 8.2% of them being undocumented (ORIM, 2021) – the share of undocumented immigrant in Italy as of January 1, 2022 is 8.4% (ISMU, 2023).

The Italian constitution safeguards health as a fundamental right of individuals regardless of their immigration status, and equitable access to health care is a core objective of the Italian National Health Service (INHS). Italy is ranked 9th out of 52 countries in terms of healthcare coverage and accessibility of services (Solano & Huddleston, 2020). Specifically, immigrants who hold a residence permit have the right and the obligation to enroll in the INHS, while undocumented immigrants are guaranteed emergency and first aid care, essential treatments and pediatrics (up to the age of 14). However, they are not entitled to register to the INHS and can only access it through an anonymous temporary code. Moreover, they cannot access General Practitioners (GPs) of the INHS – see Allegri et al. (2022) for more details on the Italian legislative framework. As a result, primary care to undocumented migrants is left to nongovernmental clinics.

Naga is one of these organizations. Based in Milan since 1987, it provides free health care to immigrants from countries of strong migratory pressure (Allegri et al., 2022) that lack health insurance in Italy and a GP in the INHS. Naga primarily serves undocumented immigrants, whereas regular migrants are readdressed to the INHS. Undocumented status is self-reported – a common, desirable practice in the field (Young & Madrigal, 2017) – and given the quality of the INHS, there is no incentive to falsely report legal status at Naga. Besides undocumented migrants, Naga's patient base also includes few citizens of countries that joined the European Union from the 2004 EU enlargement onwards (henceforth “New EU countries”). Despite being entitled to legally reside in Italy (thus being documented), they may be uninsured, due to the lack of the European Health Insurance Card needed to access to the INHS (European Parliament, 2016, p. 571375). On average, Naga's volunteer doctors carry out around 10,000 visits in a year, with new patients accounting for approximately one-fifth of this total.

## 2 Data and method

### 2.1 Data

Our study uses a unique dataset collected by Naga during 2017–18, which consists of two complementary sections. The first section is compiled by the reception staff in electronic format upon an immigrant's arrival at Naga's outpatient clinic. It includes key demographic information such as sex, age, educational background, country of origin, date of arrival in Italy, current and previous labor market conditions, living arrangements, and family and marital status (it does not specify whether family members reside in Italy or abroad). Additionally, this section records whether the individual has been a victim of torture and abuses in their home country or during transit. This information, which is not updated after the first visit, has been previously used to study undocumented immigrants' access to primary care (Devillanova, 2008) and the labor market impacts of amnesty programs (Devillanova et al., 2018).

The second section of the archive contains the medical diagnosis recorded by Naga's medical staff after each examination. These diagnoses were documented on paper up to 2020. However, for the years 2017 and 2018, one of the authors (AS) transcribed the initial visit diagnoses into electronic format, coding them using the 3-digit International Statistical Classification of Diseases and Related Health Problems, 10th Revision (ICD-10) (WHO, 2016). We do not have information on comorbidity.

We retrieved individual records for years 2017 and 2018 on November 18th, 2019. Unfortunately, medical information for the year 2019 is not available in electronic format. It is noteworthy that, starting from February 2020, Naga observed a significant reduction in the number of visits following the outbreak of the COVID-19 pandemic. This resulted in a discernible selection bias in medical cases (Devillanova et al., 2020), making more recent data unsuitable for the present study.

### 2.2 Measuring occupational downgrading

Naga's archive reports information on individuals' labor market status (inactive, unemployed, and employed) at the date of their first medical visit at Naga and before migrating. Employed individuals are also asked about their exact occupation both in Italy and in their country of origin.

To measure occupational downgrading, we first codified the occupations held by migrants, whether in Italy or their home country, using the International Standard Classification of Occupations (ISCO-08) (ILO, 2012). Subsequently, each occupation was linked to the corresponding socioeconomic categories of the *European Socio-economic Classification* (ESEC) (Harrison & Rose, 2006, p. 22), adapted to ISCO-08 b y Ganzeboom and Treiman (2010), using the *iscogen* STATA module (Jann, 2019). In line with Stringhini et al. (2017), the nine distinct ESEC categories (Harrison & Rose, 2006, p. 22) were aggregated into three broader groups: *high* (ESEC classes 1 to 3); *intermediate* (ESEC classes 4 to 6); and *low* (ESESC classes 7 to 9). Due to the very limited number of migrants in our sample who occupied positions in Italy classified as “*high*” or “*intermediate*” status, these two categories were combined into a single “*high*” category. Unemployed migrants were classified as a third category.

We operationalize occupational downgrading in the following instances: if an individual moved a) from high to low status occupation, or b) from employment (regardless of the related occupational status) to unemployment.

### 2.3 Study sample

The initial sample, referred to as the “whole sample”, consists of all the 3873 individuals aged 14 or older who had their first medical visit at Naga clinic in 2017–2018. Given the aim of the study, we restrict the analysis to the subset of individuals arrived in Italy at the age of 18 or older (323 observation excluded). To compute occupational downgrading, we have to restrict the sample to active individuals for which we can measure occupational status both before and after migration. We thus excluded individuals who were either outside the labor force (n = 117) or lacked information on their labor market status (n = 201) or current occupation (n = 64) in Italy. Similarly, individuals who were inactive (n = 670) or had unknown labor market status (n = 1097) in their home country were removed. Those with missing data on other individual characteristics used in the multivariate regression analysis, such as education (n = 189), date of arrival in Italy (n = 207), and marital status (n = 76), were also excluded. The analysis retains citizens from New EU countries, even though they cannot be undocumented, to maximize the sample size. We address the consequence of this choice in the sensitivity checks. The total number of records excluded, considering overlaps, were 2135 and the resulting regression sample includes 1738 individuals.

The main body of the text focuses on the results and analyses centered on the regression sample. However, in light of the inherent scarcity of available data on undocumented immigrants, the online appendix is thoughtfully included, offering relevant socioeconomic and medical insights derived from the whole Naga sample.

### 2.4 Statistical analysis

We estimate multivariate logistic regression models of the probability of mental and behavioral disorders of any type (ICD-10 diagnostic group V). The predictor variable of interest is *occupational downgrading*, an indicator equal to one if the immigrant experienced a loss in her/his labor market status, as previously defined, and zero otherwise.

The other controls, chosen based on a comprehensive review of pertinent literature and the availability of corresponding information in our dataset, were categorized into three domains: predetermined individual characteristics, ethnic density, and additional individual characteristics. Predetermined individual characteristics encompass: sex, age at arrival (4 age brackets: 18–34 – the excluded category –; 35–44; 45–64; 65 and more), areas of origin (Latin America —reference category—, Sub-Saharan Africa, North Africa, Asia and Europe), educational attainment (no/primary, lower secondary – reference category –, upper secondary, university), and an indicator equal to one if individual suffered torture before arrival.

‘Ethnic density’ is the (logarithm of the) number of Naga patients from the same country of origin in the initial sample. This measure is commonly employed as a proxy for the potential availability of social contacts, which can play a significant role in the development and persistence of certain types of mental health disorders (Bhugra & Jones, 2001). It is essential to note that in this context, no causal interpretation can be inferred (Blume et al., 2011). Unfortunately, we were unable to measure social isolation using an accommodation-crowding index, as done in previous studies (Fakhoury et al., 2021), due to the scarcity of information, which is available for only 380 individuals.

Finally, the three additional individual characteristics are marital status (married – the reference category –, unmarried, separated and widowed); labor market status (either unemployed or employed – reference category) and years of permanence in Italy (5-time brackets; arrived within one year is the reference category). It is important to note that those individual characteristics, although potentially relevant for an individual's mental health, are likely to be subject to reverse causality. For example, while labor market status can contribute to mental health problems, the presence of mental health issues can simultaneously reduce an individual's likelihood of obtaining or maintaining employment, thereby increasing the probability of unemployment.

Data preparation and statistical analysis was performed using Stata 18. We report adjusted odd ratios (ORs) and 95% robust confidence intervals (CIs).

## 3 Results

### 3.1 Sociodemographic characteristics

Table 1 presents the sociodemographic characteristics within the regression sample. The average age at the time of migration is 33.6 years, with males constituting 60.13% of the sample. Nearly half of the individuals are unmarried. In terms of educational attainment, 49.72% have completed at least upper secondary education, while 10.59% possess a university degree. Conversely, 19.62% of the sample either have no formal education (6.16%) or have completed only elementary education (13.46%). Regarding the duration of residence in Italy, 35.85% of the sample have lived there for less than a year, while 28.65% have resided in Italy for more than 5 years. The majority of individuals originate from Latin America, representing 31.19% of the total, with Peru being the most common country of origin, accounting for 17.26% of the sample. European migrants make up 13.81% of the sample, with 34.58% of them originating from New EU countries. This implies that 4.78% of the sample are from New EU countries, with the remaining 95.22% consisting of undocumented migrants. Notably, 50 individuals have experienced torture either in their home country or during their journey to Italy.

**Table 1:** Sociodemographic characteristics.

	No.	%		No.	%
**Sex**			**Labor market condition in Italy**		
Female	693	39.87	Employed*, of which*	620	35.67
Male	1045	60.13	*high occupational status*	6	0.97
**Age at arrival**			*low occupational status*	614	99.03
18-34	1024	58.92	Unemployed	1118	64.33
35-44	400	23.01	Occupation in Italy, if employed		
45-64	285	16.40	*Domestic helpers and cleaners*	210	33.87
65 and more	29	1.67	*Helpers and cleaners not domestic*	105	16.94
**Marital status**			*Street vendors and related*	68	10.97
Married	685	39.41	*Waiters and bartenders*	51	8.23
Unmarried	840	48.33	*Manufacturing labourers*	46	7.42
Separated	170	9.78	*Transport and freight handlers*	35	5.65
Widowed	43	2.47	*Mining and construction labourers*	26	4.19
**Education**			*Other occupations*	79	12.74
No education and primary	341	19.62	**Labor market condition at origin**		
Lower secondary	533	30.67	Employed*, of which*	1603	92.23
Upper secondary	680	39.13	*high occupational status*	366	22.83
University	184	10.59	*low occupational status*	1237	77.17
**Years since migration**			Unemployed	135	7.77
1	623	35.85	Occupation at origin, if employed		
2	279	16.05	*Shop salesperson*	363	22.65
3	203	11.68	*Clerks*	134	8.36
4	135	7.77	*Manufacturing labourers*	109	6.80
5 or more	498	28.65	*Agricultural, fisheries, and related*	95	5.93
**Region of origin**			*Teaching professionals*	84	5.24
Europe*, of which*	240	13.81	*Mining and construction labourers*	78	4.87
*New-EU*	83	34.58	*Motor-vehicle drivers*	76	4.74
*Other European countries*	157	65.42	*Other personal service*	54	3.37
Asia	302	17.38	*Cooks*	51	3.18
North Africa	417	23.99	*Machinery mechanics and fitters*	50	3.12
Sub-Saharan Africa	237	13.64	*Domestic helpers and cleaners*	47	2.93
Latin America	542	31.19	*Textile, garment, and related trades*	44	2.74
**Victim of tortures**			*Nursing and midwifery associate professionals*	37	2.31
No	1688	97.12	*Other occupations*	381	23.77
Yes	50	2.88	**Occupational downgrading**		
			No	580	33.37
			Yes	1158	66.63
			**Total**	1738	100.00

The descriptive analysis reveals that only 35.67% of individuals in the sample are employed and almost all of them work in low-status occupations (99.03%). In fact, we suspect that the six workers with a high occupational status signal a measurement error and we drop them in a later sensitivity check. Labor market outcomes in Italy contrast dramatically with those in the country of origin, where 92.23% of the sample held a job before migrating, with a non-trivial share of high-status occupations (22.83%). It follows that around two third of the study population experienced occupational downgrading. The table further list the main occupations in Italy and in the country of origin (the category “other occupations” gathers together those occupations with less than 2% of occurrences). Two features stand out in the table. Firstly, “other occupations” account for 23.77% of occupations in the country of origin compared to 12.74% in Italy. Indeed, while migrants in Italy tend to be clustered into a small number of occupations (n = 28), before migrating they have access to greater variety of job types (n = 67). Secondly, 8 out of 9 occupations in Italy (4 out of 13 in the country of origin) belong to the ISCO-08 major group 9 “Elementary Occupations”, corroborating the drop in occupational status that individuals experience upon arrival.

Appendix Table A1 presents the same sociodemographic characteristics for the whole sample. Differences between Table 1 and Table A1 are explained by the fact that the regression sample is restricted to active individuals in the working age population. Table A1 also provides additional context to the discussion on the selection criteria in section 2.2.

### 3.2 Clinical data

For each patient, the data provide the primary diagnosis assessed by the Naga medical staff. Table 2 presents this information, categorized by ICD-10 diagnostic chapters, sorted in descending order of frequency. ICD-10 chapter with few occurrences (<4% of the sample) have been grouped into a residual category. Within each ICD-10 chapter, the table also shows the three most frequent diagnoses (2-digit ICD-10 codes).

**Table 2:** Prevalence of medical diagnoses by ICD-10 chapters.

ICD-10 diagnostic group	Female		Male		Total	
*of which*	No.	%	No.	%	No.	%
**X Diseases of the respiratory system**	**56**	**8.08**	**133**	**12.73**	**189**	**10.87**
*J11 Influenza, virus not identified*	*17*	*30.36*	*45*	*33.83*	*62*	*32.80*
*J31 Chronic rhinitis, nasopharyngitis and pharyngitis*	*19*	*33.93*	*39*	*29.32*	*58*	*30.69*
*J40 Bronchitis, not specified as acute or chronic*	*7*	*12.50*	*12*	*9.02*	*19*	*10.05*
**XXI Factors influencing health status and contact with health services**	**108**	**15.58**	**80**	**7.66**	**188**	**10.82**
*Z48 Other surgical follow-up care*	*3*	*2.78*	*34*	*42.50*	*37*	*19.68*
*Z30 Contraceptive management*	*37*	*34.26*	*0*	*0.00*	*37*	*19.68*
*Z01 Other special examinations and investigations of persons without complaint or reported diagnosis*	*32*	*29.63*	*0*	*0.00*	*32*	*17.02*
**XIX Injury, poisoning and certain other consequences of external causes**	**37**	**5.34**	**135**	**12.92**	**172**	**9.90**
*S40 Superficial injury of shoulder and upper arm*	*12*	*32.43*	*31*	*22.96*	*43*	*25.00*
*T92 Sequelae of injuries of upper limb*	*5*	*13.51*	*22*	*16.30*	*27*	*15.70*
*T47 Poisoning by agents primarily affecting the gastrointestinal system*	*2*	*5.41*	*22*	*16.30*	*24*	*13.95*
**XIII Diseases of the musculoskeletal system and connective tissue**	**51**	**7.36**	**116**	**11.10**	**167**	**9.61**
*M54 Dorsalgia*	*2*	*3.92*	*5*	*4.31*	*7*	*4.19*
*M25 Other joint disorders, not elsewhere classified*	*2*	*3.85*	*5*	*4.20*	*7*	*4.09*
*M17 Gonarthrosis [arthrosis of knee]*	*20*	*39.22*	*36*	*31.03*	*56*	*33.53*
**XI Diseases of the digestive system**	**44**	**6.35**	**120**	**11.48**	**164**	**9.44**
*K30 Functional dyspepsia*	*12*	*27.27*	*38*	*31.67*	*50*	*30.49*
*K08 Other disorders of teeth and supporting structures*	*1*	*2.27*	*19*	*15.83*	*20*	*12.20*
*K64 Haemorrhoids and perianal venous thrombosis*	*6*	*13.64*	*13*	*10.83*	*19*	*11.59*
**XIV Diseases of the genitourinary system**	**109**	**15.73**	**44**	**4.21**	**153**	**8.80**
*N39 Other disorders of urinary system*	*11*	*10.09*	*20*	*45.45*	*31*	*20.26*
*N76 Other inflammation of vagina and vulva*	*28*	*25.69*	*0*	*0.00*	*28*	*18.30*
*N63 Unspecified lump in breast*	*16*	*14.68*	*0*	*0.00*	*16*	*10.46*
**XII Diseases of the skin and subcutaneous tissue**	**39**	**5.63**	**91**	**8.71**	**130**	**7.48**
*L23 Allergic contact dermatitis*	*14*	*35.90*	*28*	*30.77*	*42*	*32.31*
*L29 Pruritus*	*5*	*12.82*	*17*	*18.68*	*22*	*16.92*
*L72 Follicular cysts of skin and subcutaneous tissue*	*5*	*12.82*	*8*	*8.79*	*13*	*10.00*
**XVIII Symptoms, signs and abnormal clinical and laboratory findings, not elsewhere classified**	**56**	**8.08**	**56**	**5.36**	**112**	**6.44**
*R10 Abdominal and pelvic pain*	*29*	*51.79*	*17*	*30.36*	*46*	*41.07*
*R07 Pain in throat and chest*	*3*	*5.36*	*8*	*14.29*	*11*	*9.82*
*R42 Dizziness and giddiness*	*5*	*8.93*	*6*	*10.71*	*11*	*9.82*
**V Mental and behavioral disorders**	**41**	**5.92**	**56**	**5.36**	**97**	**5.58**
*F41 Other anxiety disorders*	12	29.27	14	25.00	26	26.80
*F43 Reaction to severe stress, and adjustment disorders*	8	19.51	11	19.64	19	19.59
*F33 Recurrent depressive disorder*	8	19.51	6	10.71	14	14.43
**IV Endocrine, nutritional, and metabolic diseases**	**47**	**6.78**	**37**	**3.54**	**84**	**4.83**
*E11 Type 2 diabetes mellitus*	*23*	*48.94*	*32*	*86.49*	*55*	*65.48*
*E03 Other hypothyroidism*	*10*	*21.28*	*0*	*0.00*	*10*	*11.90*
*E04 Other nontoxic goitre*	*4*	*8.51*	*1*	*2.70*	*5*	*5.95*
**IX Diseases of the circulatory system**	**32**	**4.62**	**49**	**4.69**	**81**	**4.66**
*I10 Essential (primary) hypertension*	*25*	*78.12*	*26*	*53.06*	*51*	*62.96*
*I87 Other disorders of veins*	*6*	*18.75*	*9*	*18.37*	*15*	*18.52*
*I48 Atrial fibrillation and flutter*	*0*	*0.00*	*11*	*22.45*	*11*	*13.58*
**VI Diseases of the nervous system**	**24**	**3.46**	**47**	**4.50**	**71**	**4.09**
*G44 Other headache syndromes*	*18*	*75.00*	*28*	*59.57*	*46*	*64.79*
*G43 Migraine*	*5*	*20.83*	*5*	*10.64*	*10*	*14.08*
*G40 Epilepsy*	*1*	*4.17*	*8*	*17.02*	*9*	*12.68*
**Other ICD-10 diagnostic groups**	**49**	**7.07**	**81**	**7.75**	**130**	**7.48**
*H60 Otitis externa*	*10*	*20.41*	*7*	*8.64*	*17*	*13.08*
*B36 Other superficial mycoses*	*2*	*4.08*	*13*	*16.05*	*15*	*11.54*
*B86 Scabies*	*2*	*4.08*	*13*	*16.05*	*15*	*11.54*
**Total**	**693**	**100**	**1045**	**100**	**1738**	**100**

Notes: For each ICD-10 chapter, the table displays the three most frequent diagnoses (2-digit ICD-10 codes).

Concerning the outcome variable of this study, 5.58% of the regression sample (equivalent to 97 individuals) received diagnoses related to mental and behavioral disorders (ICD-10 chapter V). The most frequent disorder encountered within this broader category is “other anxiety disorder” (ICD-10 code F41) (n = 26), followed by “reaction to severe stress, and adjustment disorders” (ICD-10 code F43) (n = 19), and “recurrent depressive disorder” (ICD-10 code F33) (n = 14).

Beyond diagnoses related to mental and behavioral disorders, Table 2 reveals that the primary reasons why migrants in the sample sought care at Naga's outpatient clinic include diseases of the respiratory system (10.87%), factors influencing health status and contact with health services (10.82%), cases of injury and other consequences of external causes (9.9%), diseases of the musculoskeletal system (9.61%), digestive system, (9,44%), of the genitourinary system (8.8%). These categories collectively account for approximately 60% of the diagnoses. The most frequently diagnosed condition is dorsalgia (M54), affecting 87 patients, followed by influenza (J11) in 62 patients, chronic rhinitis (J31) in 58 cases, and gonarthrosis (M17) in 56 instances. It is worth noting that preventive care is prevalent among women, for whom the two most common reasons for clinic visits are: generic contraceptive management (two-digit ICD-10 code Z30), with 37 women seeking this service; and other special examinations and investigations of persons without complaints or reported diagnoses (ICD-10 code Z01), with 32 women seeking gynecological examinations (three-digit ICD-10 code Z01.4). Preventive health examinations are less frequent among men, for whom the most common diagnoses were dorsalgia (ICD-10 code M54; 60 patients) and influenza (ICD-10 code J11; 45 cases).

Appendix Table A2 reports clinical data for the whole Naga sample. Inspection of Table 2 and Table A2 reveals no significant pattern of selection into the regression sample related to clinical conditions. Notably, the percentage of mental and behavioral disorders is slightly higher in the whole sample (6.25% vs. 5.58%).

### 3.3 Regression analysis

**Fig. 1:**
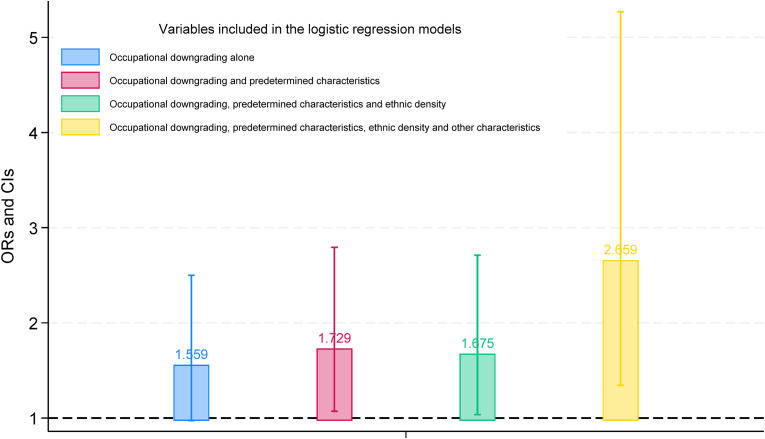
Adjusted odd ratios (ORs) and 95% confidence intervals (CIs) for mental and behavioral disorders of immigrants that experienced occupational downgrading Notes: Each bar represents the results of multivariate logistic regression models that vary in the number of controls, as detailed in the legend.

Fig. 1 displays ORs and 95% CIs for the likelihood of mental and behavioral disorders among immigrants who have experienced occupational downgrading compared to those who have not. Each bar in the figure represents the results of multivariate logistic regression models that only differ in the number of controls. The figure demonstrates that occupational downgrading is consistently associated with a heightened probability of being diagnosed with mental or behavioral disorders. In the most saturated model, the OR for occupational downgrading increases to 2.659 (95% CI 1.342–5.271), primarily due to the inclusion of individuals' labor market status. When excluding labor market status, the OR for occupational downgrading – not shown – closely aligns with that of the other three specifications (1.675, 95% CI 1.021–2.748). In summary, these results underscore that undocumented immigrants facing occupational downgrading are at a significantly elevated risk of experiencing mental and behavioral disorders.

Appendix Table A3 provides the complete estimation results. They indicate that individuals with higher levels of education are less likely to receive a diagnosis of a mental health disorder. The likelihood of psychiatric illnesses positively correlates with factors such as being unmarried, having experienced torture, and longer permanence in Italy. Conversely, it is negatively associated with ethnic density. Notably, unemployment is linked to a reduced probability of experiencing mental health issues (OR 0.532; 95% CI 0.279–1.013).

Table 3 presents the results of four sensitivity checks. First, our operationalization of Crollard et al. (2012)'s definition of occupational downgrading includes individuals that are unemployed in the host country, associating a low socioeconomic status to unemployment. We thus replicated the analyses restricting the sample to employed (column 1) and unemployed (column 2) individuals. Second, to maximize the sample size, we retain in the regression sample citizens from New EU countries. In column 3, we narrowed the sample to undocumented immigrants only, excluding individuals from new EU countries. Finally, in column 4 we replaced ethic density with a whole set of country-of-origin fixed effect, to flexibly capture differences across countries of origin that go beyond ethnic density, such as cultural traits, level of development, political condition. Due to multicollinearity, we lost 155 observations in this analysis. Results confirm the positive association between undocumented immigrants' occupational downgrading and their probability of experiencing mental and behavioral disorders. The association peaks when we clean for any country specific fixed effect (OR 3.251; 95% CI 1.451–7.283) and is most pronounced in the analysis of employed individuals (column 1).

**Table 3:** Sensitivity checks: Adjusted odd ratios and 95% confidence intervals for mental and behavioral disorders of immigrants that experienced occupational downgrading.

VARIABLES	Employed only	Unemployed only	Undocumented only	Country fixed effect
Occupational downgrading	2.515**	1.902	2.492**	3.251***
	(1.071–5.906)	(0.548–6.601)	(1.204–5.154)	(1.451–7.283)
Individual characteristics	Yes	Yes	Yes	Yes
Ethnic density	Yes	Yes	Yes	No
Observations	606	1118	1655	1583

Notes: ***, ** and * denote P values smaller than 0·01, 0·05 and 0·1, respectively. 95% robust CIs in parenthesis.

In a set of unreported results, we i) included information on housing conditions, which is imprecisely collected, and ii) dropped from the sample the six individuals with high occupational status in Italy, suspecting that they are measurement error. The ORs remained unchanged in both cases. Furthermore, we explored potential heterogeneous effects across predetermined characteristics. Although the point estimates suggest that the association between mental health issues and occupational downgrading is particularly pronounced for older workers (ages 45–64), with having a university degree appearing to mitigate this effect, these interaction terms produced imprecise OR estimates that did not achieve statistical significance.

## 4 Discussion and conclusions

This study represents a pioneering effort in quantifying the relationship between occupational downgrading and mental health among undocumented immigrants. The analysis leverages a uniquely large sample of undocumented immigrants in Milan, Italy, which includes objective medical assessments of health and a direct measure of occupational status and downgrading. The prevalence of mental conditions in the sample is 5.58%, in line with the findings of Jackson et al. (2018) that use, as we do, a medical evaluation of individuals’ health. The findings from logistic regression analyses indicate a significant correlation between occupational downgrading and a higher likelihood of receiving a diagnosis of mental or behavioral disorders.

Concerning the other variables included in the analysis, results confirm a positive association between mental conditions and lower education (Bracke et al., 2013), and social isolation (Abbas et al., 2018; Fakhoury et al., 2021); similar findings are found for refugees (Wu et al., 2021). The association between education and mental conditions suggests that highly educated individuals have more psychosocial resources to cope with the psychosocial stressors they face, as in the sample higher education is not associated to a higher socioeconomic status (Niemeyer et al., 2019). The study also highlights a positive association with longer permanence in Italy and being employed. This latter result, together with the results obtained for the restricted sample of employed individuals, leads to believe that the high prevalence of 3-D jobs (Moyce & Schenker, 2018) in this population has adverse effects on mental health, suggesting also that occupational downgrading affects immigrants’ mental health beyond and above the unfavorable work conditions they face. It should also be noted that if there is selection out of employment of workers with poor mental health conditions, our result would underestimate the effect of employment as a stress factor.

The paper provides unique insights into the sociodemographic characteristics and health of this often hard-to-reach population. The analysis underscores the poor integration outcomes observed in the sample, with a low number of employed individuals typically engaged in low-status occupations.

Despite the richness of the Naga archive, it does have limitations. A crucial caveat is that it is a selected sample of migrants seeking medical care at a single outpatient clinic in Milan (Devillanova, 2008), warning on the generalizability of the results to other contexts. This limitation is common in studies on undocumented migrants, mainly based on convenience non-representative sample data (Garcini et al., 2021), with exceptions (Allegri et al., 2022; Gimeno-Feliu et al., 2020). Individual characteristics, except for medical diagnoses, are self-reported and may be subject to recall bias, as well as challenges related to language barriers. Neither can we rule out undiagnosed mental conditions in patients seeking care for other issues. Finally, missing observations for certain variables preclude their inclusion in the analysis (e.g. crowding index). It is important to note that the cross-sectional data used in this study cannot distinguish between age, age at arrival, and length of stay, possibly hiding a higher prevalence of mental disorders in older individuals (Andersson et al., 2018).

From a policy perspective, the results highlight the importance of considering the impact of policies beyond those linked to the healthcare system, including restrictive entry and integration policies, on migrant health (Juárez et al., 2019; Vernice et al., 2020). For undocumented migrants, barriers to access the labor market and occupational downgrading do have mental health consequences that add to the other stressors that they constantly face (Garcini et al., 2021). These factors contribute to the formation of social inequalities between native and foreign-born groups. Importantly, if both social causation and social selection/drift (Lund & Cois, 2018) act simultaneously – i.e. if low/downgraded occupational status affects mental health conditions and poor mental health hampers labor market integration – these effects are likely to persist over time. Furthermore, they are unlikely to be confined in the population of undocumented immigrants, due to the substantial mobility across legal statuses (Spencer & Triandafyllidou, 2022). These aspects should always be taken into consideration in the design of sound migration policies. Finally, we advocate for access to primary care for all immigrants, in order to timely detect and early treat mental conditions in populations of undocumented immigrants, ultimately breaking the cycle of poor labor market outcomes and poor mental health in this highly vulnerable group.

## Declaration of interests

We declare no competing interests.

## Funding

The authors received no specific funding for this work.

## Disclaimer (legal notice)

The opinions expressed are those of the author(s) only and should not be considered as representative of the European Commission's official position. This article does not involve the European Commission in liability of any kind.

## Ethics approval

All procedures performed in studies involving human participants were in accordance with the ethical standards of the institutional and/or national research committee and with the 1964 Helsinky Declaration and its later amendments or comparable ethical standards. The research obtained ethical approval from the Ethics Committee of Bocconi University, #FA000093.

## CRediT authorship contribution statement

**Carlo Devillanova:** Writing – review & editing, Writing – original draft, Methodology, Investigation, Formal analysis, Conceptualization. **Cristina Franco:** Writing – review & editing, Writing – original draft, Formal analysis. **Anna Spada:** Data curation.

## Data Availability

The authors do not have permission to share data.
