# Supplementary material for: Downgraded dreams: Labor market outcomes and mental health in undocumented migration
Source: SSM Popul Health. 2024 Mar 6;26:101652. doi: 10.1016/j.ssmph.2024.101652 (PMC10950686; doi:10.1016/j.ssmph.2024.101652)
Supplement: Multimedia component 1 [file mmc1.doc]

# Downgraded dreams: Labor market outcomes and mental health in undocumented migration

***Appendix tables***

Table A 1: Sociodemographic statistics of individuals included in the whole Naga sample

|  | | **No.** | **%** |  | | | **No.** | | **%** | |
| --- | --- | --- | --- | --- | --- | --- | --- | --- | --- | --- |
| **Sex** | |  |  | **Labor market condition in Italy** | | | |  | |  |
|  | Female | 1586 | 40.95 |  | Employed*, of which* | | | 1257 | | 32.46 |
|  | Male | 2287 | 59.05 |  |  | *high occupational status* | | *19* | | *1.51* |
| **Age at arrival** | |  |  |  |  | *low occupational status* | | *1158* | | *92.12* |
|  | 0-18 | 323 | 8.34 |  |  | *Unknown* | | 80 | | 6.36 |
|  | 18-34 | 2037 | 52.59 |  | Unemployed | | | 2298 | | 59.33 |
|  | 35-44 | 652 | 16.83 |  | Inactive | | | 117 | | 3.02 |
|  | 45-64 | 512 | 13.22 |  | Unknown | | | 201 | | 5.19 |
|  | 65 and more | 349 | 9.01 |  | Occupation in Italy, if employed | | |  | |  |
| **Marital status** | |  |  |  | | Domestic helpers and cleaners | | 333 | | 26.49 |
|  | Married | 1396 | 36.04 |  | | Helpers and cleaners not domestic | | 218 | | 17.34 |
|  | Unmarried | 1982 | 51.17 |  | | Street vendors and related | | 139 | | 11.06 |
|  | Separated | 302 | 7.80 |  | | Waiters and bartenders | | 112 | | 8.91 |
|  | Widowed | 117 | 3.02 |  | | Manufacturing labourers | | 111 | | 8.83 |
|  | Unknown | 76 | 1.96 |  | | Transport and freight handlers | | 69 | | 5.49 |
| **Education** | |  |  |  | | Mining and construction labourers | | 43 | | 3.42 |
|  | No education and primary | 794 | 20.50 |  | | Other occupations | | 168 | | 13.37 |
|  | Lower secondary | 1199 | 30.96 |  | | Unknown | | 64 | | 5.09 |
|  | Upper secondary | 1345 | 34.73 | **Labor market status at origin** | | | |  | |  |
|  | University | 346 | 8.93 |  | Employed*, of which* | | | 1938 | | 50.04 |
|  | Unknown | 189 | 4.88 |  |  | *high occupational status* | | *428* | | *22.08* |
| **Years since migration** | |  | |  |  | *low occupational status* | | *1510* | | *77.92* |
|  | 1 | 1321 | 34.11 |  | Unemployed | | | 168 | | 4.34 |
|  | 2 | 530 | 13.68 |  | Inactive | | | 670 | | 17.30 |
|  | 3 | 393 | 10.15 |  | Unknown | | | 1097 | | 28.32 |
|  | 4 | 255 | 6.58 |  | Occupation at origin, if employed | | |  | |  |
|  | 5 or more | 1167 | 30.13 |  | | Shop salesperson | | 419 | | 21.62 |
|  | Unknown | 207 | 5.34 |  | | Clerks | | 152 | | 7.84 |
| **Region of origin** | |  |  |  | | Manufacturing labourers | | 142 | | 7.33 |
|  | Europe*, of which* | 586 | 15.13 |  | | Agricultural, fisheries, and related | | 132 | | 6.81 |
|  | *New-EU* | *361* | *61.60* |  | | Teaching professionals | | 101 | | 5.21 |
|  | *Other European countries* | *225* | *38.40* |  | | Mining and construction labourers | | 97 | | 5.01 |
|  | Asia | 629 | 16.24 |  | | Motor-vehicle drivers | | 91 | | 4.70 |
|  | North Africa | 964 | 24.89 |  | | Other personal service | | 68 | | 3.51 |
|  | Sub-Saharan Africa | 564 | 14.56 |  | | Cooks | | 67 | | 3.46 |
|  | Latin America | 1130 | 29.18 |  | | Machinery mechanics and fitters | | 59 | | 3.04 |
| **Victim of tortures** | |  |  |  | | Domestic helpers and cleaners | | 58 | | 2.99 |
|  | No | 3772 | 97.39 |  | | Textile, garment, and related trades | | 51 | | 2.63 |
|  | Yes | 101 | 2.61 |  | | Nursing and midwifery associate professionals | | 40 | | 2.06 |
|  |  |  | |  | | Other occupations | | 461 | | 23.79 |
|  |  |  | | **Occupational downgrading** | | | |  | |  |
|  |  |  | |  | No | | | 658 | | 16.99 |
|  |  |  | |  | Yes | | | 1310 | | 33.82 |
|  |  |  | |  | Unknown | | | 1905 | | 49.19 |
|  |  |  | | **Total** | | | | **3873** | | **100.00** |

*Notes: The whole sample comprises all the 3,873 uninsured/undocumented migrants (90.67% of which are undocumented) who that had their first visit at Naga outpatient clinic, Milan, Italy, during 2017-18.*

Table A 2: Prevalence of medical diagnoses by ICD-10 chapters in individuals included in the whole Naga sample

| **ICD-10 diagnostic group** | | **Female** | | **Male** | | **Total** | |
| --- | --- | --- | --- | --- | --- | --- | --- |
|  | *of which* | *No.* | *%* | *No.* | *%* | *No.* | *%* |
| **XXI Factors influencing health status and contact with health services** | | **231** | **14.56** | **210** | **9.18** | **441** | **11.39** |
|  | *Z48 Other surgical follow-up care* | *6* | *2.60* | *87* | *41.43* | *93* | *21.09* |
|  | *Z02 Examination and encounter for administrative purposes* | *10* | *4.33* | *79* | *37.62* | *89* | *20.18* |
|  | *Z30 Contraceptive management* | *83* | *35.93* | *0* | *0.00* | *83* | *18.82* |
| **X Diseases of the respiratory system** | | **123** | **7.76** | **304** | **13.29** | **427** | **11.03** |
|  | *J31 Chronic rhinitis, nasopharyngitis and pharyngitis* | *43* | *34.96* | *88* | *28.95* | *131* | *30.68* |
|  | *J11 Influenza, virus not identified* | *39* | *31.71* | *89* | *29.28* | *128* | *29.98* |
|  | *J40 Bronchitis, not specified as acute or chronic* | *12* | *9.76* | *38* | *12.50* | *50* | *11.71* |
| **XIII Diseases of the musculoskeletal system and connective tissue** | | **137** | **8.64** | **239** | **10.45** | **376** | **9.71** |
|  | *M54 Dorsalgia* | *63* | *45.99* | *126* | *52.72* | *189* | *50.27* |
|  | *M17 Gonarthrosis [arthrosis of knee]* | *60* | *43.80* | *78* | *32.64* | *138* | *36.70* |
|  | *M25 Other joint disorders, not elsewhere classified* | *8* | *5.84* | *10* | *4.18* | *18* | *4.79* |
| **XI Diseases of the digestive system** | | **106** | **6.68** | **234** | **10.23** | **340** | **8.78** |
|  | *K30 Functional dyspepsia* | *35* | *33.02* | *82* | *35.04* | *117* | *34.41* |
|  | *K08 Other disorders of teeth and supporting structures* | *6* | *5.66* | *40* | *17.09* | *46* | *13.53* |
|  | *K64 Haemorrhoids and perianal venous thrombosis* | *10* | *9.43* | *25* | *10.68* | *35* | *10.29* |
| **XIX Injury, poisoning and certain other consequences of external causes** | | **67** | **4.22** | **270** | **11.81** | **337** | **8.70** |
|  | *S40 Superficial injury of shoulder and upper arm* | *25* | *37.31* | *66* | *24.44* | *91* | *27.00* |
|  | *T92 Sequelae of injuries of upper limb* | *8* | *11.94* | *39* | *14.44* | *47* | *13.95* |
|  | *T47 Poisoning by agents primarily affecting the gastrointestinal system* | *2* | *2.99* | *38* | *14.07* | *40* | *11.87* |
| **XIV Diseases of the genitourinary system** | | **234** | **14.75** | **83** | **3.63** | **317** | **8.18** |
|  | *N39 Other disorders of urinary system* | *25* | *10.68* | *35* | *42.17* | *60* | *18.93* |
|  | *N76 Other inflammation of vagina and vulva* | *57* | *24.36* | *0* | *0.00* | *57* | *17.98* |
|  | *N63 Unspecified lump in breast* | *36* | *15.38* | *1* | *1.20* | *37* | *11.67* |
| **XII Diseases of the skin and subcutaneous tissue** | | **92** | **5.80** | **206** | **9.01** | **298** | **7.69** |
|  | *L23 Allergic contact dermatitis* | *33* | *35.87* | *62* | *30.10* | *95* | *31.88* |
|  | *L29 Pruritus* | *11* | *11.96* | *36* | *17.48* | *47* | *15.77* |
|  | *L72 Follicular cysts of skin and subcutaneous tissue* | *11* | *11.96* | *22* | *10.68* | *33* | *11.07* |
| **XVIII Symptoms, signs and abnormal clinical and laboratory findings, not elsewhere classified** | | **115** | **7.25** | **129** | **5.64** | **244** | **6.30** |
|  | *R10 Abdominal and pelvic pain* | *61* | *53.04* | *40* | *31.01* | *101* | *41.39* |
|  | *R07 Pain in throat and chest* | *7* | *6.09* | *23* | *17.83* | *30* | *12.30* |
|  | *R05 Cough* | *8* | *6.96* | *16* | *12.40* | *24* | *9.84* |
| **V Mental and behavioural disorders** | | **100** | **6.31** | **142** | **6.21** | **242** | **6.25** |
|  | *F41 Other anxiety disorders* | *33* | *33.00* | *35* | *24.65* | *68* | *28.10* |
|  | *F43 Reaction to severe stress, and adjustment disorders* | *15* | *15.00* | *23* | *16.20* | *38* | *15.70* |
|  | *F33 Recurrent depressive disorder* | *20* | *20.00* | *17* | *11.97* | *37* | *15.29* |
| **IX Diseases of the circulatory system** | | **103** | **6.49** | **101** | **4.42** | **204** | **5.27** |
|  | *I10 Essential (primary) hypertension* | *73* | *70.87* | *49* | *48.51* | *122* | *59.80* |
|  | *I87 Other disorders of veins* | *12* | *11.65* | *19* | *18.81* | *31* | *15.20* |
|  | *I48 Atrial fibrillation and flutter* | *12* | *11.65* | *19* | *18.81* | *31* | *15.20* |
| **IV Endocrine, nutritional and metabolic diseases** | | **114** | **7.19** | **84** | **3.67** | **198** | **5.11** |
|  | *E11 Type 2 diabetes mellitus* | *60* | *52.63* | *64* | *76.19* | *124* | *62.63* |
|  | *E03 Other hypothyroidism* | *18* | *15.79* | *4* | *4.76* | *22* | *11.11* |
|  | *E66 Obesity* | *11* | *9.65* | *3* | *3.57* | *14* | *7.07* |
| **VI Diseases of the nervous system** | | **70** | **4.41** | **93** | **4.07** | **163** | **4.21** |
|  | *G44 Other headache syndromes* | *47* | *67.14* | *57* | *61.29* | *104* | *63.80* |
|  | *G40 Epilepsy* | *7* | *10.00* | *18* | *19.35* | *25* | *15.34* |
|  | *G43 Migraine* | *13* | *18.57* | *7* | *7.53* | *20* | *12.27* |
| **Other ICD-10 diagnostic groups** | | **94** | **5.93** | **192** | **8.40** | **286** | **7.38** |
|  | *B36 Other superficial mycoses* | *5* | *5.32* | *29* | *15.10* | *34* | *11.89* |
|  | *H60 Otitis externa* | *15* | *15.96* | *17* | *8.85* | *32* | *11.19* |
|  | *B86 Scabies* | *4* | *4.26* | *26* | *13.54* | *30* | *10.49* |
| **Total** | | **1586** | **100** | **2287** | **100** | **3873** | **100** |

*Notes: The whole sample comprises all the 3,873 uninsured/undocumented migrants (90.67% of which are undocumented) that had their first visit at Naga outpatient clinic, Milan, Italy, during 2017-18. The diagnosis pertains to the first medical consultation, and the ICD-10 chapters are arranged in descending order of frequency. Diagnostic chapters with limited occurrences (<4%) have been consolidated into a residual category. For each ICD-10 chapter, the* *table displays the three most frequent diagnoses (2-digit ICD-10 codes).*

Table A 3: Adjusted odd ratios and 95% confidence intervals for mental and behavioral disorders

|  | *(1)* | *(2)* | *(3)* | *(4)* |
| --- | --- | --- | --- | --- |
|  |  |  |  |  |
| Occupational downgrading | 1.559* | 1.729** | 1.675** | 2.659*** |
|  | (0.972 - 2.500) | (1.071 - 2.793) | (1.035 - 2.711) | (1.342 - 5.271) |
| Male |  | 0.695 | 0.702 | 0.670 |
|  |  | (0.424 - 1.139) | (0.425 - 1.159) | (0.391 - 1.147) |
| Age at arrival 35-44 |  | 0.780 | 0.774 | 1.073 |
|  |  | (0.455 - 1.338) | (0.452 - 1.327) | (0.594 - 1.937) |
| Age at arrival 45-64 |  | 0.682 | 0.689 | 1.073 |
|  |  | (0.358 - 1.303) | (0.358 - 1.326) | (0.519 - 2.221) |
| Age at arrival 65 and more |  | 0.553 | 0.594 | 1.455 |
|  |  | (0.073 - 4.174) | (0.077 - 4.586) | (0.152 - 13.961) |
| No education and primary education |  | 0.544* | 0.556* | 0.598 |
|  |  | (0.292 - 1.015) | (0.296 - 1.045) | (0.314 - 1.141) |
| Upper secondary |  | 0.545** | 0.542** | 0.515*** |
|  |  | (0.336 - 0.886) | (0.334 - 0.880) | (0.312 - 0.849) |
| University |  | 0.345** | 0.305*** | 0.247*** |
|  |  | (0.145 - 0.823) | (0.127 - 0.731) | (0.100 - 0.611) |
| Europe |  | 1.493 | 1.256 | 1.545 |
|  |  | (0.750 - 2.974) | (0.617 - 2.557) | (0.742 - 3.218) |
| Asia |  | 1.787* | 1.286 | 1.505 |
|  |  | (0.926 - 3.446) | (0.635 - 2.605) | (0.719 - 3.151) |
| North Africa |  | 1.139 | 1.314 | 1.406 |
|  |  | (0.535 - 2.426) | (0.607 - 2.844) | (0.627 - 3.152) |
| Sub-Saharan Africa |  | 1.408 | 0.931 | 1.073 |
|  |  | (0.644 - 3.078) | (0.403 - 2.149) | (0.470 - 2.448) |
| Victim of tortures |  | 5.552*** | 6.144*** | 5.587*** |
|  |  | (2.526 - 12.204) | (2.759 - 13.682) | (2.428 - 12.853) |
| Ethnic density |  |  | 0.752*** | 0.742*** |
|  |  |  | (0.632 - 0.895) | (0.619 - 0.889) |
| Unmarried |  |  |  | 2.555*** |
|  |  |  |  | (1.475 - 4.425) |
| Separated |  |  |  | 1.098 |
|  |  |  |  | (0.462 - 2.609) |
| Widowed |  |  |  | 0.458 |
|  |  |  |  | (0.043 - 4.885) |
| Years since migration 2 |  |  |  | 1.041 |
|  |  |  |  | (0.556 - 1.949) |
| Years since migration 3 |  |  |  | 1.292 |
|  |  |  |  | (0.653 - 2.556) |
| Years since migration 4 |  |  |  | 2.099** |
|  |  |  |  | (1.044 - 4.218) |
| Years since migration 5 or more |  |  |  | 1.073 |
|  |  |  |  | (0.579 - 1.987) |
| Unemployed |  |  |  | 0.532* |
|  |  |  |  | (0.279 - 1.013) |
| Constant | 0.043*** | 0.059*** | 0.270** | 0.132*** |
|  | (0.029 - 0.065) | (0.032 - 0.110) | (0.090 - 0.815) | (0.034 - 0.518) |
|  |  |  |  |  |
| Observations | 1,738 | 1,738 | 1,738 | 1,738 |
| Robust cieform in parentheses |  |  |  |  |
| *** p<0.01, ** p<0.05, * p<0.1 |  |  |  |  |

*Notes: ORs for the probability of mental and behavioral disorders of any type (ICD-10 diagnostic group V). The table reports complete results of the four multivariate logistic regression models shown in figure 1 of the main text. As for the figure, each specification varies in the number of controls: occupational downgrading alone (column 1); occupational downgrading and predetermined characteristics (column 2); occupational downgrading, predetermined characteristics and ethnic density (column 3); occupational downgrading, predetermined characteristics, ethnic density and other characteristics (column 4).***, ** and * denote P values smaller than 0·01, 0·05 and 0·1, respectively. 95% robust CIs in parenthesis.*
